# Declaration on infection prevention and management in global surgery

**DOI:** 10.1186/s13017-023-00526-3

**Published:** 2023-12-06

**Authors:** Massimo Sartelli, Federico Coccolini, Luca Ansaloni, Walter L. Biffl, David P. Blake, Marja A. Boermeester, Raul Coimbra, Heather L. Evans, Paula Ferrada, George Gkiokas, Marc G. Jeschke, Timothy Hardcastle, Chandler Hinson, Francesco M. Labricciosa, Sanjay Marwah, Antonio C. Marttos, Martha Quiodettis, Kemal Rasa, Jianan Ren, Ines Rubio-Perez, Robert Sawyer, Vishal Shelat, Jeffrey S. Upperman, Fausto Catena

**Affiliations:** 1 Department of Surgery, Macerata Hospital, Via Santa Lucia 2, 62100 Macerata, Italy; 2 General, Emergency and Trauma Surgery Department, Pisa University Hospital, Pisa, Italy; 3 Department General Surgery, Fondazione IRCCS San Matteo, Pavia, Italy; 4 Scripps Memorial Hospital La Jolla, La Jolla, CA USA; 5 Division of Acute Care Surgery, Inova Health System, Falls Church, VA USA; 6 University of Virginia School of Medicine, Charlottesville, VA USA; 7 USUHS/F Edward Hebert School of Medicine, Bethesda, MD USA; 8 Department of Surgery, Amsterdam University Medical Centre, Amsterdam, The Netherlands; 9 Division of Trauma and Acute Care Surgery, Riverside University Health System Medical Center, Moreno Valley, CA USA; 10 Department of Surgery, Medical University of South Carolina, Charleston, SC USA; 11 Second Department of Surgery, Aretaieion University Hospital, National and Kapodistrian University of Athens, 10679 Athens, Greece; 12 Hamilton Health Sciences, Hamilton, ON Canada; 13 Department of Health - KwaZulu-Natal, Surgery, University of KwaZulu-Natal and Inkosi Albert Luthuli Central Hospital, Durban, South Africa; 14 Frederick P. Whiddon College of Medicine, University of South Alabama, Mobile, AL USA; 15 Global Alliance for Infections in Surgery, Macerata, Italy; 16 Department of Surgery, BDS Post-Graduate Institute of Medical Sciences, Rohtak, India; 17 Department of Surgery, University of Miami Miller School of Medicine, Miami, USA; 18 Division of Trauma and Acute Care Surgery, Hospital Santo Tomas, Panama City, Panama; 19 Department of Surgery, Anadolu Medical Center, Kocaeli, Turkey; 20 Research Institute of General Surgery, Jinling Hospital, Affiliated Hospital of Medical School, Nanjing University, Nanjing, China; 21 Unit of Colorectal Surgery, Department of General Surgery, Hospital Universitario La Paz, Madrid, Spain; 22 Department of Surgery, Western Michigan University Homer Stryker MD School of Medicine, Kalamazoo, MI USA; 23 Department of General Surgery, Tan Tock Seng Hospital, Singapore, Singapore; 24 Department of Pediatric Surgery, Vanderbilt Children’s Medical Center, Nashville, TN USA; 25 General and Emergency Surgery, Bufalini Hospital, Cesena, Italy

**Keywords:** Antibiotic therapy, Antimicrobial resistance, Education, Hospital-acquired infections, Infection prevention and control, Surgery, Surgical antibiotic prophylaxis, Surveillance

## Abstract

Surgeons in their daily practice are at the forefront in preventing and managing infections. However, among surgeons, appropriate measures of infection prevention and management are often disregarded. The lack of awareness of infection and prevention measures has marginalized surgeons from this battle. Together, the Global Alliance for Infections in Surgery (GAIS), the World Society of Emergency Surgery (WSES), the Surgical Infection Society (SIS), the Surgical Infection Society-Europe (SIS-E), the World Surgical Infection Society (WSIS), the American Association for the Surgery of Trauma (AAST), and the Panamerican Trauma Society (PTS) have jointly completed an international declaration, highlighting the threat posed by antimicrobial resistance globally and the need for preventing and managing infections appropriately across the surgical pathway. The authors representing these surgical societies call all surgeons around the world to participate in this global cause by pledging support for this declaration for maintaining the effectiveness of current and future antibiotics.

## Introduction

Despite evidence supporting the effectiveness of best practices in preventing and managing infections, many surgeons worldwide fail to implement them, and evidence-based practices tend to be underused in routine clinical practice.

Although most surgeons are aware of the problem of antimicrobial resistance (AMR), many still underestimate this problem in their own hospitals. Inappropriate use of antibiotics, as well as poor respect of infection prevention and control measures such as hand hygiene, is contributing to the development of AMR [1]. Surgeons are at the forefront of preventing and managing infections and should take a proactive role in ensuring the avoidance of inappropriate and unnecessary antibiotic use in hospital settings [2].

## Methods

Together, the Global Alliance for Infections in Surgery (GAIS), the World Society of Emergency Surgery (WSES), the Surgical Infection Society (SIS), the Surgical Infection Society-Europe (SIS-E), the World Surgical Infection Society (WSIS), the American Association for the Surgery of Trauma (AAST), and the Panamerican Trauma Society (PTS) have shared an international declaration, highlighting the threat posed by AMR globally and the need for preventing and managing infections appropriately across the surgical pathway. As such, it is its intent to raise global awareness among surgeons about AMR focusing on the importance of participating in this challenge.

This declaration has been signed by a task force of expert physicians representing the seven surgical societies involved in the alliance. They participated in the realization of the definitive document.

A global declaration on the appropriate use of antibiotics across the surgical pathway signed by a multidisciplinary working group was published in 2017 [2]. Differently from that declaration this one, signed by some of the scientific societies most involved in surgical infections and addressed to surgeons, focuses on their crucial role in preventing and managing infections to contribute to the fight against AMR.

## Declaration on infection prevention and management in global surgery

Beginning with the discovery of penicillin by Alexander Fleming in the late 1920s, antibiotics have revolutionized modern medicine saving millions of lives each year. However, bacteria have developed resistance to antibiotics, causing infections that are more serious and difficult to treat.

Antibiotics can be lifesaving when managing patients with bacterial infections. However, they are often used inappropriately, unnecessary administered for long durations, or used without consideration of pharmacokinetic and pharmacodynamic principles.

Large variability in antibiotic prescriptions exists among countries worldwide, and while excessive use remains a major problem in some regions of the world, elsewhere there is a lack of access to many antibiotics. This disparity creates a concerning gap jeopardizing the sustainability and safety of the global antibiotic supply, ultimately compromising access to effective treatments and leading to suboptimal prescription practices in some areas of the world. Effective antibiotic treatment is an essential component of delivering universal healthcare, and the whole world has a collective responsibility to use antibiotics appropriately to maintain their effectiveness [1].

AMR is a natural phenomenon occurring as microorganisms evolve; however, human activities are accelerating the pace at which bacteria develop and spread AMR. Inappropriate antimicrobial prescribing practices, as well as incorrect infection prevention and control measures, are contributing to the development and spread of AMR. AMR is a complex issue involving not only humans but also animals and the environment. The term "One Health" is now accepted to define the strict interconnectedness of the health of people, animals, and the environment where we live [3].

Hospitalized patients often have multiple risk factors for the acquisition of AMR. Acute care facilities are incubators for the spread of AMR representing an environment that can facilitate the emergence and the spread of resistant organisms. Healthcare workers in hospital settings play a critical role in preventing the spread of AMR. In 2008, the acronym “ESKAPE” including *Enterococcus faecium, Staphylococcus aureus, Klebsiella pneumoniae, Acinetobacter baumannii, Pseudomonas aeruginosa,* and *Enterobacter* species was coined to emphasize that these bacteria currently cause the majority of hospital infections and can “escape” the effects of antibiotics [4].

Alarming levels of AMR have been reported in all countries, regardless of their average income level [2]. On the basis of predictive statistical models, Murray and collaborators [4] estimated 4.95 million deaths associated with bacterial AMR in 2019, including 1.27 million deaths attributable to bacterial AMR. The six leading bacterial pathogens associated with AMR were *Escherichia coli, followed by Staphylococcus aureus, Klebsiella pneumoniae, Streptococcus pneumoniae, Acinetobacter baumannii, and Pseudomonas aeruginosa*. These bacteria were responsible for 929,000 deaths attributable to AMR and 3.57 million deaths associated with AMR in 2019 [4].

Although antibiotic resistance is a widely recognized public health threat, less is known about the burden of antifungal resistance. The frequency of fungal infections has raised in recent years, largely because of the increasing size of the at-risk population, which includes patients with cancer, transplant recipients, immunocompromised patients such as patients with human immunodeficiency virus infection, and other patients who receive immunosuppressive therapy. In addition, fungal infections are relatively common in critically ill patients and are associated with considerable morbidity and death. Recently, an insidious multidrug-resistant species, *Candida auris*, has emerged causing persistent multi-regional outbreaks [1].

Of significant importance in limiting AMR is efforts to prevent hospital-acquired infections (HAIs). Patients with medical devices (central lines, urinary catheters, and ventilators) or who undergo surgical procedures are at high risk of acquiring HAIs. Prevention is better than cure, and every infection prevented is one that does not need treatment. Infection prevention and control can be cost-effective and implemented everywhere, even where resources are limited. HAIs result in significant morbidity and mortality, prolong the duration of hospital stay, and may necessitate additional diagnostic and therapeutic interventions.

**Fig. 1:**
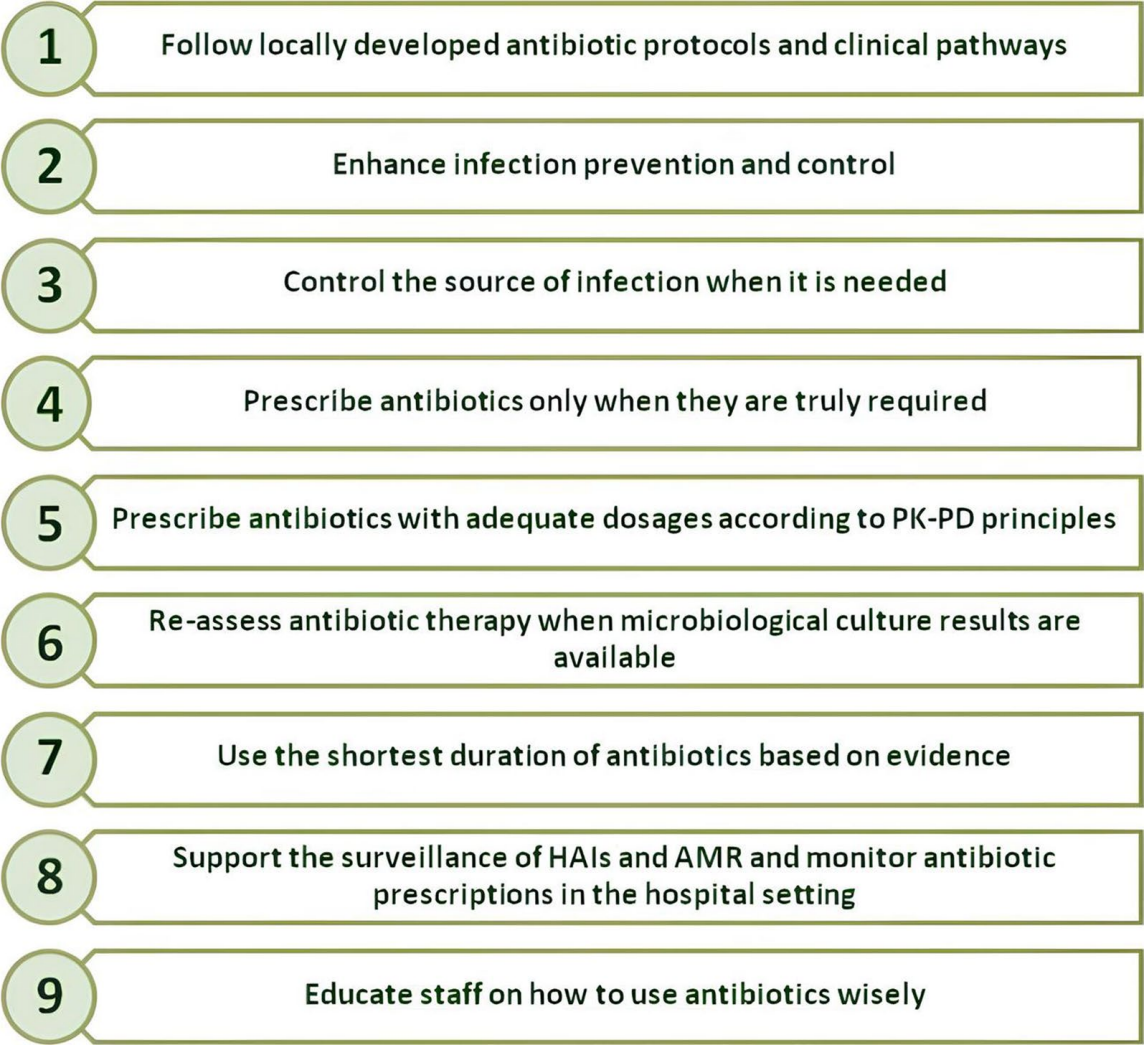
Fundamental principles for correct infection prevention and management across the surgical pathway [2]. HAIs: healthcare-associated infections. AMR: antimicrobial resistance. PK–PD: pharmacokinetic–pharmacodynamic

Physicians in hospital settings should be aware of their role and responsibility in maintaining the effectiveness of current and future antibiotics. In Fig. 1, the fundamental principles for correct infection prevention and management across the surgical pathway [2] are illustrated.

Surgeons in their daily practice are at the forefront in preventing and managing infections [5]. They are responsible for many of the processes that impact the risk for surgical site infections and play a key role in their prevention. Surgical antibiotic prophylaxis (SAP) plays a pivotal role in perioperative infection prevention and control. The use of SAP remarkably contributes to the total amount of antibiotics used in hospitals and may be correlated to increases in AMR and healthcare costs. Surgeons are also at the forefront in managing patients with infections, who often need prompt source control and appropriate antibiotic therapy, and are directly responsible for their outcomes.

However, among surgeons, appropriate infection prevention and control measures and antibiotic prescribing practices are often disregarded [1]. In hospitals, cultural, contextual, and behavioural determinants can influence surgeons daily clinical practice, and a range of factors such as the fear of clinical failure, time pressure, or organizational contexts can limit the adherence to infection prevention and control measures and antibiotic prescribing practices. Moreover, because of cognitive dissonance (recognizing that action is necessary but not implementing it), changing clinical behaviour is extremely challenging [1]. The lack of awareness of these measures has distanced surgeons from this fight. In many hospitals around the world, surgeons are not involved in antimicrobial stewardship programmes, even if they are prescribers of antibiotics both for prophylaxis and therapy. Furthermore, surgeons are often not involved in infection prevention and control programmes, even if they are primarily responsible for preventing hospital-acquired infections, particularly surgical site infections.

Collaboration is essential in providing care that is appropriate to meet the needs of patients and optimize individual patients’ outcomes and overall healthcare delivery. A collaborative approach allows each member of the team to participate and to be responsible for their respective contributions to patient care. Understanding the importance of preventing and managing infections across the surgical pathway involves the creation of a culture of collaboration in which infection prevention and control, antimicrobial stewardship, and optimal surgical approaches are all closely interconnected with each other. In the context of a multidisciplinary approach, on which modern medicine is based, the direct leadership of surgeons in preventing and managing infections is of utmost importance.

If surgeons around the world participate in this global fight, they will be pivotal leaders in addressing this global challenge. Otherwise, they will be contributors to the worst crisis that the world health is facing.

## Conclusion

The authors call all surgeons around the world to participate in this global cause by pledging support for this declaration and accepting responsibility for maintaining the effectiveness and longevity of current and future antibiotic agents.

Now is the time to act!

## Data Availability

Not applicable.

## References

[1] Sartelli M, Duane TM, Catena F, Tessier JM, Coccolini F, Kao LS (2016). Antimicrobial stewardship: a call to action for surgeons. Surg Infect (Larchmt).

[2] Global Alliance for Infections in Surgery Working Group (2017). A global declaration on appropriate use of antimicrobial agents across the surgical pathway. Surg Infect (Larchmt).

[3] World Health Organization. Antimicrobial resistance. https://www.who.int/health-topics/antimicrobial-resistance. Accessed 16 Oct 2023.

[4] Antimicrobial Resistance Collaborators (2022). Global burden of bacterial antimicrobial resistance in 2019: a systematic analysis. Lancet.

[5] Sartelli M, Weber DG, Ruppé E, Bassetti M, Wright BJ, Ansaloni L (2016). Antimicrobials: a global alliance for optimizing their rational use in intra-abdominal infections (AGORA). World J Emerg Surg.

